# Impact on place of death in cancer patients: a causal exploration in southern Switzerland

**DOI:** 10.1186/s12904-020-00664-4

**Published:** 2020-10-15

**Authors:** Heidi Kern, Giorgio Corani, David Huber, Nicola Vermes, Marco Zaffalon, Marco Varini, Claudia Wenzel, André Fringer

**Affiliations:** 1 Triangolo Association, In Sceresòra 4, CH-6528 Camorino, Switzerland; 2 IDSIA, Dalle Molle Institute for Artificial Intelligence, Galleria 2, Via Cantonale 2c, CH-6928 Manno, Switzerland; 3 Department Health Sciences (Institute for Therapeutic Sciences), KREMS, IMC University of Applied Sciences, Piaristengasse 1, A-3500 Krems, Austria; 4 Department Health, Institute of Nursing, Zurich University of Applied Sciences ZHAW, Katharina-Sulzer-Platz 9, Postfach, CH-8401 Winterthur, Switzerland

**Keywords:** Cancer, End-of-life care, Palliative home care, Place of death, Communication, Family caregivers, Patient preference, Bayesian networks, Credal networks, Bayesian classifiers

## Abstract

**Background:**

Most terminally ill cancer patients prefer to die at home, but a majority die in institutional settings. Research questions about this discrepancy have not been fully answered. This study applies artificial intelligence and machine learning techniques to explore the complex network of factors and the cause-effect relationships affecting the place of death, with the ultimate aim of developing policies favouring home-based end-of-life care.

**Methods:**

A data mining algorithm and a causal probabilistic model for data analysis were developed with information derived from expert knowledge that was merged with data from 116 deceased cancer patients in southern Switzerland. This data set was obtained via a retrospective clinical chart review.

**Results:**

Dependencies of disease and treatment-related decisions demonstrate an influence on the place of death of 13%. Anticancer treatment in advanced disease prevents or delays communication about the end of life between oncologists, patients and families. Unknown preferences for the place of death represent a great barrier to a home death. A further barrier is the limited availability of family caregivers for terminal home care. The family’s preference for the last place of care has a high impact on the place of death of 51%, while the influence of the patient’s preference is low, at 14%. Approximately one-third of family systems can be empowered by health care professionals to provide home care through open end-of-life communication and good symptom management. Such intervention has an influence on the place of death of 17%. If families express a convincing preference for home care, the involvement of a specialist palliative home care service can increase the probability of home deaths by 24%.

**Conclusion:**

Concerning death at home, open communication about death and dying is essential. Furthermore, for the patient preference for home care to be respected, the family’s decision for the last place of care seems to be key. The early initiation of family-centred palliative care and the provision of specialist palliative home care for patients who wish to die at home are suggested.

## Background

Studies report that a majority of cancer patients prefer to die at home [1–5]. Despite longstanding research concerning the improvement and implementation of policies favouring end-of-life (EOL) home care, most patients die in institutional settings. An incongruence of approximately 20% between patients’ preferred place of death (POD) and patients’ actual POD has been reported worldwide [6]. Based on the calculated eight million deaths from cancer every year, it must be assumed that approximately 1.6 million cancer patients cannot die at their preferred location. The trajectory of cancer diseases is usually characterised by a steady progression and a short phase of a clear decline prior to death that lasts weeks or months [7]. These characteristics lead to the hypothesis that the approaching terminal phase of the disease might be predictable and that EOL care planning could be initiated in time to meet the patient’s wishes and preferences. Assessing preferences for the last place of care prior to death and for the POD requires challenging conversations among health care professionals, patients, and family members [8]. In the advanced stages of the disease, when patients are undergoing anticancer treatment, these conversations often occur too late to enable preferences to be turned into reality [9]. This trend contradicts the hypothesis of the predictability of death and the ability for advance care planning. It must be assumed that preferences are often unknown until the phase of imminent death. The aim of the project is to identify predictors and favourable patterns for home death, as well as to examine the possibilities of interventions provided by healthcare professionals to facilitate home-based EOL. Within this context, the study explores the question of which variables and dependencies between variables can be identified with respect to the POD and what knowledge can be generated for health care professionals involved in cancer care to better support the patient’s wish to die at home.

## Methods

This study was a retrospective chart review in a regional cancer centre in the Italian-speaking part of southern Switzerland. It explored the interplay of a wide spectrum of factors influencing cancer patients’ POD, such as disease-, individual-, family-, care network- and policy-related factors. For this purpose, two research software tools were developed: a causal probabilistic model for data analysis and knowledge (a *credal network*) [10–12] and a data mining algorithm (a *classifier*) [13].

Classifiers are one of the main tools used in machine learning; they are statistical methods that learn from examples. In our application each example is a pair made by information about a patient and the related POD (including at home, in a hospital or a nursing home). After being fed with a number of such examples, a classifier learns by itself the relation between the former and the latter; whence, after the learning stage, it can be employed to predict the POD of a patient given the available information about such a patient. The classifier used in our study is one of the most well-known and with a long history of successful applications: the so-called *naive Bayes classifier* [14]. We stress that our classifier was created on the sole basis of empirical data as reported in Table 1. We measured its predictive accuracy compared to that of the patient’s care team, and they turned out to be equivalent.

**Table 1:** Patient demographic and clinical characteristics and caregiver and care network characteristics

Characteristics	Details characteristics	Total *n* = 116
Time interval between assessment and death (days)	Mean	38
	Range	3–89
Gender	Male	52
	Female	64
Age(years)	20–40	1
	41–65	33
	66–80	58
	> 80	24
Primary tumour site	Gatrointestinal tract	40
	Lung	12
	Breast	15
	All other tumours	48
Time interval between diagnosis and death (days)	Mean	922
	Range	2–10,465
Place of death	Home	21
	Hospital	90
	Nursing home	5
Time interval between last administration of chemotherapy and death (days)	Mean	34
	Range	0–247
Distance from residence to cancer centre (km)	Mean	4
	Range	1–74
Area of residence	Rural	31
	Urban	85
Living arrangements	Alone	40
	With spouse	65
	With siblings	2
	With children	6
	Other	3
Visits from palliative physicians	Yes	35
	No	81
Visits from palliative nurses	Yes	93
	No	23
Social worker	Involved	49
	Not involved	67
Volunteers	Involved	71
	Not involved	45
General practitioner	Home visits	39
	No home visits	54
	Not available	23
Home care services	Public home care service	33
	Private home care service	41
	None provided	42
Continuous home care provided by migrant care workers	Provided	3
Place of care in the 60 days prior to assessment (range 0–60)		
Days spent at home	Mean	48
Days spent in a hospital	Mean	10
Days spent in a nursing home	Mean	0
No. of hospitalisations in the 60 days prior to assessment	Mean	1
	Range	0–3
Karnofsky Performance Status	10–40	41
	50–60	63
	70–100	12
Symptom burden	None-low	6
	Medium	36
	High	74
Cancer treatment	Ongoing	67
	Discontinued	49
Patient’s awareness of dying	Open	81
	Closed	34
	Not assessed	1
Patient’s preference for place of death	Home	34
	Hospital	9
	Nursing home	1
	Not assessed	72
Family’s awareness of dying	Open	87
	Closed	18
	Not assessed	11
Family system’s conditions for home care	Suitable	39
	Unsuitable	75
	Not assessed	2
Family’s preference for place of death	Home	29
	Hospital	20
	Nursing home	1
	Not assessed	66
Care team’s prediction of place of death	Home	39
	Nursing home	2
	Hospital	75

Data obtained by clinical chart review (median 38 days before death)

The second tool that we have employed is a probabilistic-graphical model called a *credal network*. The goal of such a model is more ambitious than that of a classifier. In fact, the network aims at representing the phenomenon in its entirety: that is, modelling all the factors together with their causal interactions, in a joint way. This enables one to do analysis that a classifier cannot do, in particular analysis of interventions: for instance, what happens to the POD if one manipulates some of the factors? If we, for instance, impose the choice of a certain treatment. This is more demanding, and arguably more important, than merely doing predictions based on passive observations, since a tool like this can help to devise new policies without trying them in reality (something that is not always ethically possible, or that is costly). Let us note that credal networks are in close relation with one of the most popular tools in Artificial Intelligence, which are called Bayesian networks. A *Bayesian network* [15] is equivalent to a joint probability distribution over the factors (variables) of the problem; it is therefore completely compatible with (Bayesian) probability theory. A credal network generalises the concept of Bayesian network to make it more reliable (robust), while still being completely compatible with probability theory. It does so by allowing incomplete knowledge to enter the model. In particular, given the large spectrum of factors to explore, large amounts of data would have been required to build the causal probabilistic model using only empirical data from patients. To compensate for a lack of evidence from the data set, a further source of information, namely, expert knowledge, was exploited. Such a knowledge is organized in a qualitative and a quantitative part. The former is provided by means of a directed graph where nodes represent factors (variables) of the problem and arcs between them represent cause-effect relations. Quantitative knowledge is provided in a probabilistic form to express the strength of the causal relations in the graph. Notably, credal nets permit such probabilistic knowledge to be coded in the model without forcing the expert to say, only for modelling convenience, more than her knowledge allows: for example, when the expert was uncapable of assessing the precise numerical probability of a certain event, the model allowed the expert to provide in its place weaker probabilistic judgments, like that one event is very probable, or more probable than another, without requiring a number. This is a necessary basis to build credible models that deliver reliable inference. The variables and states of variables used for the causal model are listed in Table 2 in Additional file 1. Connections between variables are represented in the directed acyclic graph presented in Fig. 1 in Additional file 2. A detailed description of the technical features is given in Additional file 3. The validation procedure consisted of retrospectively testing the predictive capacity.

Let us stress finally that both, the classifier and the credal network, allow one to reason on specific patients: they are not only a tool for analysis of population. This is made possible by their underlying probabilistic nature: whenever one focuses on a new patient, the system (be it the classifier or the credal net) is fed with information about that very patient, and the inference engine yields prediction for that very patient as a consequence of the application of Bayes conditioning.

The research tools can be accessed online: http://ipg.idsia.ch/software.php?id=141.

### Data source and setting

Data were gathered in a regional cancer centre from a total of 116 adult patients who died from cancer between 2015 and 2016 in southern Switzerland. The three most frequent cancer diagnoses were of the gastrointestinal tract (34%), breast (13%) and lung (10%). Seventy-eight percent of the patients died in a hospital, 18% died at home and 4% died in a nursing home.

Routine data on patients’ demographic and clinical characteristics were sampled retrospectively through clinical chart review at a median time of 38 days (range 3–89 days) prior to death. The data set is given in Table 1, and the collection procedure is presented in Table 3 in Additional file 4.

All research procedures were submitted to the Cantonal Ethics Committee of Ticino. Given the retrospective study design, no informed consent from patients or caregivers could be obtained. Ethical approval and consent were obtained in September 2016 under the protocol number Swissethics ID BASEC 2016–01455.

## Results

The results mentioned in this section were obtained via queries with the two research tools, mainly the causal probabilistic model. Various scenarios of daily clinical practice were simulated by testing the impact of the variables and their different states on a target variable. The output of the model was calculated in the form of probability intervals with lower and upper values. Therefore, some of the results are expressed ranges. The remainder of the results are expressed as percentages, which were calculated based on differences in the lower values of the probability interval since a higher prevalence is attributed to variations in the lower value. The results from these queries are detailed in Table 4 and Table 5 in Additional file 5.

### Results from the causal probabilistic model

#### Dependencies of disease and treatment-related variables

Among illness-related variables, the following dependencies were observed. As long as effective anticancer treatment resources are available, these resources are most likely to be used to treat metastatic cancer until near the EOL, with a 64–80% likelihood of use for this purpose, with the goal of palliating symptoms and improving survival. Ongoing cancer treatment prevents or delays communication about EOL issues between oncologists, patients and families. Ongoing cancer treatment decreases the probability of open communication by 40% compared with that of treatment discontinuation. In the investigated study population, the last chemotherapy was administered at a median of 34 days before death. Of the patients, 60 to 80% were probably only partially informed about the proximity of death when undergoing anticancer treatments. Thus, patients and their relatives are more likely to remain in a closed rather than open state of awareness of dying. When patients are not aware of the terminal stage of the disease, they probably do not communicate about their preferences for the place of care and POD. Accordingly, assessing preferences for the EOL period is difficult when patients and families are not ready to discuss the subject. In the current study, these preferences were unknown among approximately two-thirds of the 116 patients at a median of 38 days prior to death. Eighty-seven percent of these patients died in a hospital.

Communication between health care professionals, patients and families about the imminence of death shows a positive impact on the awareness context of 40%. Most patients and their relatives (60–80%) shift from a closed to an open state of awareness of dying following open communication.

In addition to the influence of ongoing anticancer treatment on communication and the awareness context, other aspects must be taken into account. As long as palliative chemotherapy is continued, the treatment of toxicities and their consequences require additional supportive care, which leads to higher rates of hospital admissions and consequently to more days spent in a hospital. The more days patients spend in a hospital, the more likely they are to die in that setting. The number of hospital days is also influenced by the symptom burden. Severe symptoms increase the probability of prolonged hospital stays. A large proportion of cancer patients suffer from a high symptom burden or unstable clinical conditions during the last weeks of life, requiring inpatient care as long as the treatment approach is active.

The interplay of these disease and treatment-related variables shows an influence on the home death rate of 13%.

#### Dependencies of patient and family preferences

For patients living at home, preferences for home as the place of care (37–63%) are slightly higher than preferences for the hospital (28–53%). Patients resident in a nursing home prefer to be cared for in that setting (57–67%), followed by home (23–31%) and then the hospital (8–14%). Family caregivers’ preferences contrast the wishes of patients, especially in urban areas and agglomerations, where family caregivers clearly prefer hospitals (48–82%) versus homes (16–49%). In rural areas, there is almost no difference in preferences for home (35–60%) versus the hospital (37–62%). The family’s preference shows the highest overall impact on POD at 51%, which is much higher than the impact of the patient’s preference, at 14%. The family’s preference is strongly dominant over the patient’s preferences. A significant congruence between the family’s preference and POD can be observed, independent of the patient’s preference: when the family’s preference is home, the probability of a home death is high (53–70%); when the family’s preference is the hospital, the patient is more likely to die in a hospital (59–87%); and when the family’s preference is a nursing home, the patient is more likely to die in a nursing home (50–79). The best probability for home death is subject to a preference for home care that is expressed by both patients and family caregivers (64–76%).

#### Dependencies of family-related variables

The family’s preference for the last place of care is mostly influenced by the family system’s conditions for home care. The probability of suitable conditions is best (80–100%) when at least one person from the family is available to assist with home care, when the family members have somewhat solid emotional relationships, when the family members are fully informed about the patient’s approaching death, when the family members have an open state of awareness, and when the patient has a low degree of dependence. Among these variables, the relevance of the state of awareness seems to be important. In a state of closed awareness, the suitability of the family’s conditions for home care drops from 80 to 100 to 60%. An even more obvious trend becomes apparent if no family member is available for home care. Then, the probability of suitable conditions decreases from 80 to 100% to 20–40%. An analogous decline from 80 to 100 to 40% is observable in the family’s availability for home care when the patient suffers from severe symptoms, has a high degree of dependence and is in need of continuous help and support.

Economic resources seem to play a role only for families without their own resources in place for home care since the families then must pay for expensive assistance from professional carers. In this event, economic resources show an impact of 20% on the family’s preference. These circumstances might be country specific. In the current study population, continuous assistance provided by health professionals or care workers with migrant backgrounds is not provided for by law and is therefore not covered by health insurance. Only three of the 116 families in the study had chosen this solution.

The interplay of all family-related variables, excluding the family’s preference, demonstrates a final impact on POD of 13%. However, a clear prediction for home death cannot be made as long as the family’s preference is unknown.

#### Dependencies of care network and policy-related variables

To support the design of policies, the impacts of the following potentially modifiable variables were tested. The use of specialist palliative home care services, access to home visits by GPs, access to home care nurses, access to volunteer hospice services and coverage of home care costs by health insurance reflect the effect of an interdisciplinary home care network and health care policy. The interplay of this group of modifiable variables demonstrates an impact on the home death rate of only 6% when patient and family preferences are unknown. In contrast, in the subgroup of patients and families with a congruent preference for home care, an interdisciplinary home care network can consistently increase the probability of home death by at least 58%. These patients actually have the highest probability of dying at home overall, at 76–83%. Without the use of specialist palliative home care services, the chance of dying at home decreases by 24%, but the likelihood of a home death is still higher than that of a hospital death, at 51–59% versus 41–50%, respectively. No difference is observed in the impact on the likelihood of a home death between routine and continuous professional home care. Continuous professional assistance does not seem to be more effective than routine home care.

As shown before, if the family’s preference for the POD is the hospital, despite full access to an interdisciplinary home care network, the probability of dying at home drops significantly by 57%, from 76 to 83% to 19–40%. This finding confirms the strong impact of the family’s preference on POD and leads to the hypothesis that if interventions by the interdisciplinary home care network could influence the family’s choice for the place of care, the home death rate would increase significantly. However, these variables reveal a low impact on the family’s preference of only 4%. As a result, the family decision-making process depends neither on the home care network nor on the health care policy but rather mainly on the family conditions for home care.

In the search for hypothetical interventions to better support family conditions, open EOL communication that improves the state of awareness and good symptom control can be identified as an option. The impact of such an intervention demonstrates an increased probability of suitable conditions for home care by 35%, yielding a final influence on the home death rate of 17%.

### Results from the classifier

The results from the classifier mainly confirm the results obtained from the causal model. A high probability of home death (97%) is observed for patients who live in a rural environment, have a low symptom burden, have spent few days in a hospital, have an open awareness of dying, have a suitable family system for home care, have congruent preferences for home care with their families, receive home care assistance provided by a home care service, have access to GP home visits and have access to specialist palliative home care. In urban areas, the probability of death at home drops slightly to 82%. Without the involvement of a specialist palliative home care service, the probability decreases significantly to 46%. When severe symptoms set in, even though all other conditions appear favourable, the probability of home death drops from 82 to 59%. In the case of a long hospital stay, the chance of death at home is only 23%. This tendency becomes even more apparent when the family’s awareness of dying is closed. Then, the probability of home death decreases to just 13%.

## Discussion

The aim of this project was to explore predictors and favourable patterns for home death. The main results of this study largely explain the reality of the low percentage of home deaths among cancer patients. With respect to the POD, two main processes can be defined as relevant. The family preference for place of care, with an influence of 51%, and dependencies on disease and treatment-related decisions, with an influence of 13%, represent the key factors.

The cause-effect relationships between the investigated variables seem to be initiated by the increasing availability of effective anticancer treatment resources. An active treatment attitude close to the dying phase delays communication about approaching death and therefore decreases the probability of conversations about EOL preferences. Several authors have reported findings that support these assertions. Difficulties in determining the adequate timing of the disclosure of imminent death [16], high burdens on oncologists when communicating treatment discontinuation [17, 18], inaccurate prediction of the survival time [19], a goal to not deprive patients of hope [18], and unrealistic expectations and requests of patients and families [20, 21] represent relevant barriers to effective EOL communication. The relationship between the timing of communication about death and the POD must be considered significant. If EOL conversations occur late, the time of the conversation will be too late to assess patient and family preferences and to prepare the family for the challenging task of home care. EOL caregiving is complex and frightening. As reported by Thomas, fear about not having experience and capacity to take care of a terminally ill person, a lack of understanding of symptom progression and management, as well as uncertainty about what might happen when death is imminent and how to deal with imminent death are known burdens in family caregivers [22]. Family members spend an average of 43 h a week providing care. When continuous assistance is necessary, spouses may provide up to 100 h per week [23]. The availability of caregiving at home drops significantly when symptoms set in and patients become completely dependent on physical care. However, the nature of most advanced cancer diseases causes severe symptoms or rapidly changing clinical conditions, which does not support a peaceful EOL period at home. Taking care of a dying relative at home can be an unstable transition, during which families struggle for normality and are challenged by changing roles and relationships and ambivalent feelings [24]. Thus, the family has to be seen as a social system in which resources might be aligned not only to the preferences of the dying family member but also to the attempt to maintain a normal life that includes jobs, childcare and other activities of a busy daily life. Key family caregiver characteristics that increase the likelihood of a home death are female gender and a high level of ability to deliver care at home for as long as necessary [25]. Furthermore, awareness about the imminent dying process is of central importance. When families lack an understanding of death, the conditions for home care remain more likely not to be suitable. Death needs to be discerned and accepted, and at least one family caregiver should assume the responsibility of allowing death to occur at home [26]. Therefore, the results of this study highlighting the strong influence of family-related variables on POD are not surprising. Ultimately, it is the family system that decides on the place of care for the dying member. The family’s choice for the place of care cannot be influenced by healthcare professionals; it mostly depends on personal resources, experiences, coping strategies and caring attitudes. Patients without family support have almost no chance for home care unless they have high economic resources for external care.

### Preferences for the place of care

Patient preferences for home as the place of care, at 37–63%, were surprisingly lower than expected in comparison with preferences for the hospital, at 28–53%, which were higher than assumed. However, these results confirm the tendency of a broadly acknowledged higher preference for a home death [3–6]. A systematic review of the United Kingdom literature highlighted a contrasting view of patient preferences, concluding that patients’ preferred POD is unknown because of large amounts of missing data and the handling of these missing data. Patients might not express their wishes regarding their POD depending on the person asking the question, the setting and the context [27]. Rainsford confirmed that wishes and preferences might not be the same. A major concern of terminally ill people is not to be a burden on their relatives, and therefore, they might express a preference for hospital care [1]. According to Pleschberger, “what is said is often not what is meant”, and perspectives can change over time [28]. A quarter of the patients with a preference for home death changed their opinions when death was imminent [6]. The patient’s wish to die at home can be based on two different meanings of home: home as a concrete living place or home as a metaphor for well-being within a benevolent environment [29]. For people living alone without any family support, the preference for home might lose its meaning because what dying patients basically need is someone to take care of them.

The nursing home seems to be a preference only for those patients who lived in a nursing home before the terminal stage of their illness (57–68%). For these patients, the nursing home has become their second home. As a second choice, they would prefer to be cared for at home, but this option is unlikely to be realistic because residents of nursing homes probably do not have a supportive family network available to take care of them at home. Preferences for the hospital can be considered low in residents of nursing homes, at 8–14%. For patients living at home, the nursing home is the least preferred place of care, at 6–14%. In the literature, data regarding the preference for a nursing home are less available than data for home and hospital preferences. The reported range of 1–12% is slightly lower than that found in the current study, but it confirms this tendency [6].

The family caregiver preferences contrast the wishes of patients, especially in urban areas and agglomerations, where the preference is clearly for hospitals, 48–81%, versus homes 16–49%. In rural areas, no difference between home and hospital can be observed. This might be explained by cultural or traditional attitudes in caregiving. A systematic review based on the results of 210 studies confirmed higher preferences for home among patients than among family caregivers [5]. The highest probability of a home death was found for patients and families with an agreed preference for home care, at 64–76%. A high congruence between the family caregiver preference and the actual POD [30], a poor congruence between patient and family preferences and the dominance of the family preference over the patient preference have been reported in other studies [31]. It can be concluded that the question of where cancer patients prefer to be cared for at the end of their lives and where they would prefer to die cannot be fully answered, but preferences for home seem to be prevalent, while family caregivers more often prefer hospital EOL care.

### Care network and health care policy

Access to an interdisciplinary home care network including specialist palliative home care services increases the number of deaths at home, but not significantly, with an increase of only 6%, in a general population of cancer patients when preferences are unknown. By contrast, an increased probability of 58% can be observed when both the patient’s and family’s preferences are for home and when they are assisted by an interdisciplinary home care network. These findings are identical to those of Costa et al., Costa and Alonso-Babarro et al. [2, 32, 33]. Specific home care programmes that include visits from GPs and home care nurses in addition to specialist palliative home care services are essential support for patients and families who prefer home as the place of care. For this constellation of conditions, the model expresses the highest probability of a home death of 76–83%. The question about home care frequency cannot yet be fully answered since it is unknown whether continuous care is effective in increasing the home death rate [34]. In the current study, no difference between routine and continuous professional home care was observed. Continuous home care seems to not be more effective than routine home care. This might be explained by a poor acceptance of external carers in this study population. Only three of 116 patients had continuous care provided by care workers with migrant backgrounds. The benefit of home care likely occurs when the care is provided by family members rather than professional carers. A care programme similar to the hospital, with nurses or other health care professionals continuously present at the home, is not what patients and families want. As Gronemeyer and Heller stated, the concept of home is lost when the hospital is outsourced to the home [35].

Regarding the family’s decision-making process for the place of care, a multidisciplinary home care team seems to have almost no influence, at 4%. The family’s choice depends mostly on their own resources and coping strategies. Open communication is a more relevant factor, as it improves the state of awareness of patients and families. Open communication and good symptom control can enhance the family system’s conditions for home care by 35%, demonstrating a final impact on POD of 17%. This percentage can be considered important in the context of an incongruence of approximately 20% between the preferred and actual POD.

### Limitations

To the best of our knowledge, this is the first attempt to comprehensively depict the complexity and causality of the POD topic through the application of state-of-the-art artificial intelligence techniques, such as classifiers and credal networks. Despite the high congruence of the reported results with those of evidence-based publications, our models have obviously also some limitations. The classifier has been inferred on the basis of a small data set. Even though its accuracy has proven competitive with that of human experts, one should not blindly trust its predictions that could, for instance, be more uncertain on some patients than on some others. We recommend collecting more data for future developments of such a predictive tool. With regard to the credal network, its main weakness, which is also part of its strength, is that it relies for a large part on expert knowledge. We have tried to minimize possible subjective biases by checking the literature with care for broadly shared knowledge about the POD problem. But of course, some bias can still be present.

The data set collected in only one cancer centre represents a further limitation, as cultural and regional effects could affect some results.

## Conclusions

Although these limitations need to be considered, we think that the main results can be of interest to health care providers in a global context, as the study reveals two crucial issues for confronting incurable cancer: difficulties in communicating about death and dying and the limited availability of EOL home care by family caregivers. The temporal domain appears to be one of the most relevant factors. The late timing of EOL conversations due to available treatment options delays the assessment of the patient’s wishes and needs. Struggling against the disease and maintaining hope seem to be frequent coping strategies. The wish to continue anticancer treatment might outweigh the desire to spend the last weeks of life at home and to die there. The question of where patients prefer to die needs to be viewed in the whole context of the circumstances characterising the last few weeks or days of life, in which some aspects might be more relevant than others. The family system plays the most important role in this context. The family’s preference shows the overall strongest impact on the POD. Therefore, to support the patient’s preference for a home death, interventions need to be directed at the family system. Home care of dying patients needs to start in a timely fashion. Early initiation of home care requires a considerable effort initiated by the oncologist as the principal communicator about treatment decisions, as well as optimal collaboration within the interdisciplinary care network and the family. Approximately one-third of family systems can be empowered by open EOL communication and good symptom control when favourable conditions are recognised at the right time. Considering that these factors might influence decision making among family systems by as much as 35%, providing specialist palliative home care that offers family-centred palliative care would be the ideal solution for all families with favourable preconditions for home care. The involvement of a specialist palliative home care team can be suggested for patients and families who express a strong preference for EOL home care.

The developed research tools can be used as a basis for the implementation of sensitivity concepts in cancer networks. The classifier performed best in prediction and might be used as an assessment tool in clinical practice. The credal net model is easily understandable to non-specialists of these methodologies, thanks to its graphical representation; for this reason, such a model could be employed as an educational tool for health care professionals to gain a deeper understanding of the causality and complexity of the topic of *dying at home.* More generally speaking, we hope that our effort to code the overall interactions between factors in the POD problem can serve as a basis for discussion, criticism, and eventually improvements of this model.

## Supplementary information

**Additional file 1 **: **Table 2**. Elicited variables of the causal probabilistic model

**Additional file 2 **: **Figure 1**. Directed acyclic graph of the causal probabilistic model

**Additional file 3.** : Technical features of the research tools

**Additional file 4 **: **Table 3**. Data collection procedure

**Additional file 5 **: **Table 4**: Results from the causal probabilistic model and **Table 5**: Results from the classifier

## Data Availability

All data supporting the findings in this study are included within the manuscript and additional files. The dataset used and/or analysed during the current study is available from the corresponding author on reasonable request.

## Abbreviations

POD: Place of death
EOL: End of life
