# Supplementary material for: Impact on place of death in cancer patients: a causal exploration in southern Switzerland
Source: BMC Palliat Care. 2020 Oct 15;19:160. doi: 10.1186/s12904-020-00664-4 (PMC7566155; doi:10.1186/s12904-020-00664-4)
Supplement: Supplementary file 1 — Additional file 1 : Table 2. Elicited variables of the causal probabilistic model [file 12904_2020_664_MOESM1_ESM.pdf]

Additional file 1. Table 2: Variables and states in the causal probabilistic model

Table 2. Elicited variables and states of variables of the causal probabilistic model	
Variables	States of variables
Core variable: Place of death	Outcome: Home; hospital; nursing home
Individual-related variables	*Individual-related states of variables*
Days spent in a hospital 60 days prior to assessment	0-20; 21-40; 41-60
Age (years)	20-40; 41-65; 66-80; >80
Karnofsky Performance Status (KPS)	0-40; 45-65; 70-100
Degree of dependence	Almost independent; needs routine help; needs continuous help
Patient's cognition	Normal cognition; mild impairment; impairment
Patient's residence	Home; nursing home
Patient's awareness of dying	Open; closed
Patient's preference for place of care	Home; nursing home; hospital
Illness-related variables	*Illness-related states of variables*
Symptom burden	None-low; medium-high
Comorbidity	Non or not severe; severe
Cancer treatment resources	Available; not available
Cancer treatment	Ongoing active treatment; treatment discontinued
Family-related variables	*Family-related states of variables*
Family's awareness of dying	Open; closed
Family's emotional relationship	Somewhat solid; somewhat conflictual
Family's availability for home care	At least one person available; nobody available
Family system's conditions	Suitable for home care; unsuitable for home care
Family's preference for place of care	Home; nursing home; hospital
Family's time resources	Poor; limited; unlimited
Geographic distance of the family caregiver	0-5 km; 5-50 km; >50 km
Area of residence	Rural; urban
Family's economic resources for home care	Poor; high
Income benefit support	No; yes
Care network-related variables	*Care network-related states of variables*
Oncologist: Communication of poor prognosis	Little information about the end of life given; partial information given; full information given
General practitioner	Home visits; no home visits
Professional home care frequency	No home care; routine home care; continuous home care
Specialist palliative home care service	Yes; no
Volunteer hospice service	Available for a few hours; available 24/7
Health care policy-related variables	*Health care policy-related states of variables*
Federal health care policy	Home care partially covered; fully covered
Health insurance	Home care costs partially covered; fully covered
