# Supplementary figures and images for: Impact on place of death in cancer patients: a causal exploration in southern Switzerland
Source: BMC Palliat Care. 2020 Oct 15;19:160. doi: 10.1186/s12904-020-00664-4 (PMC7566155; doi:10.1186/s12904-020-00664-4)

Additional file 2: Figure 1. Directed acyclic graph of the causal probabilistic model

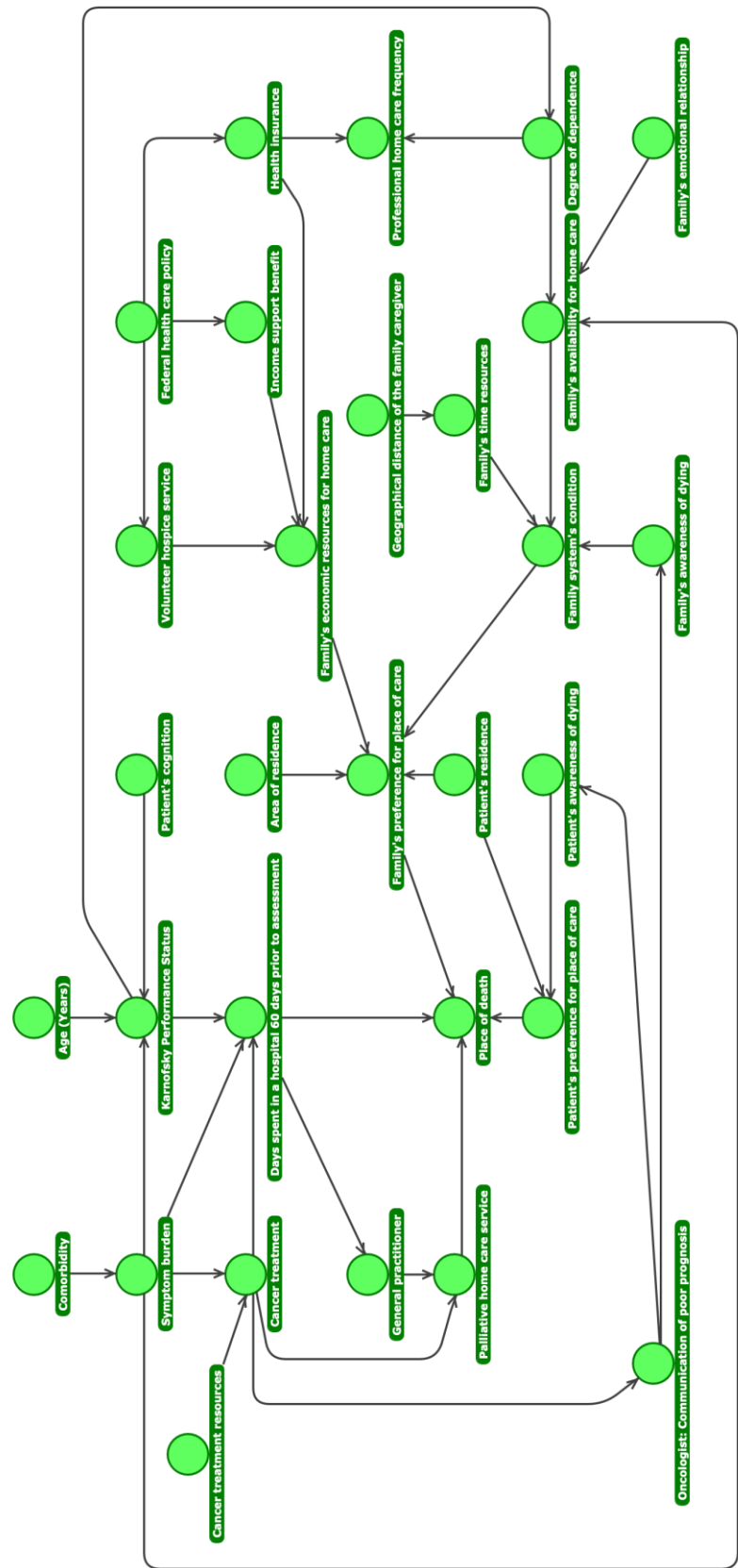

Supplement: Supplementary file 2 — Additional file 2 : Figure 1. Directed acyclic graph of the causal probabilistic model [file 12904_2020_664_MOESM2_ESM.pdf]
