# Supplementary material for: Impact on place of death in cancer patients: a causal exploration in southern Switzerland
Source: BMC Palliat Care. 2020 Oct 15;19:160. doi: 10.1186/s12904-020-00664-4 (PMC7566155; doi:10.1186/s12904-020-00664-4)
Supplement: Supplementary file 3 — Additional file 3. : Technical features of the research tools [file 12904_2020_664_MOESM3_ESM.pdf]

Technical Appendix

1 Introduction

Our contribution is a probabilistic causal model of the factors affecting the place of death. We start by eliciting from experts the cause-effect relations between more than 30 variables and we represent them through a directed acyclic graph (DAG). In the Bayesian paradigm, a prior distribution if first elicited based on domain knowledge, and it is then updated to incorporate the evidence extracted from the data. As a first step, we allow experts to specify in natural language the conditional probabilities of each children given its parents within the DAG. We then translate these qualitative assessments in a probabilistic assessment using the methodology of [1]. In order to model the vagueness of the language assessments, we define a set of prior distributions rather than a sharp single prior distribution for each node of the DAG. A model of this type is called a *credal network* [4]; credal networks have already been used for representing expert knowledge in the military field [1] and for geological risk assessment [2].

We validate the model by testing (retrospectively) its predictions on 116 cases of terminally-ill patients in southern Switzerland. The predictions of the credal network compare favorably to both the predictions of a team of experts. and to those of different data mining approaches. As a result of being based on a set of distributions, on some hard-to-classify instances the credal network returns a set of classes. On such hard-to-classify instances both the other data mining approaches and the team of experts undergo a sharp drop of accuracy. Instead the credal network remains reliable by highlighting the unpredictability of such instances and then returning a set of classes.

2 The causal graph

We elicit the cause - effect relations between more than 30 variables relevant to the place of death. The result is the directed acyclic graph of Fig.1. All variables are discrete.

The core variable is *place of death*, which has three states: *home*, *nursing home*, *hospital*. We synthetically describe in the following the model, focusing on the variables which are closest to *place of death* within the DAG.

Place of death has four parents:

- number of *days spent in hospital* (0-20, 21-40, 41-60) in the 60 days before assessment;
- *patients preferred place of death* (home, hospital, nursing home);
- *family's preferred place of death* (home, hospital, nursing home);
- availability of a *palliative home-care* service (yes, no).

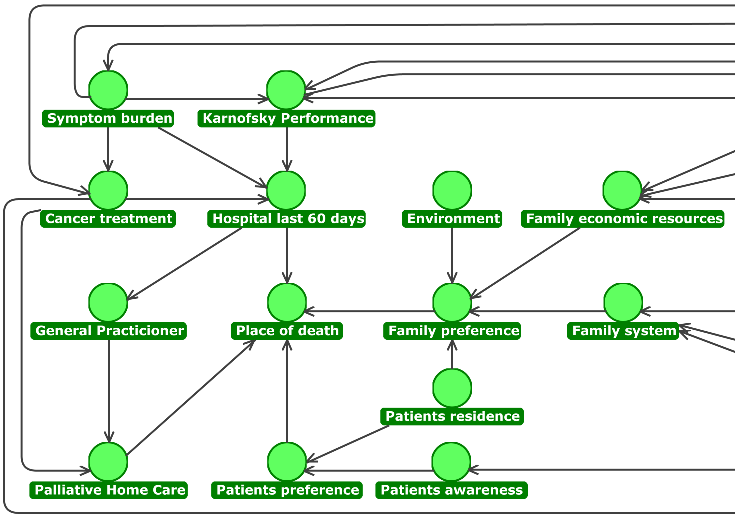

Fig. 1: Zoom-in of the DAG around *place of death*.

The *number of days spent in hospital* in the last 60 days depends on the cancer-related symptoms, on the type of cancer treatment (ongoing treatment, treatment interruption) and on the functional status of the patient. The functional status of the patient is assessed through the Karnofsky performance status (0-45, 46-65, 66-100), which is affected both by the patients age (20-40, 41-65, 66-80, >80) and by his cognitive state (impairment, mild impairment, no impairment).

The *patient preferred place of death* (home, hospital, nursing home) depends on where he/her current resides (at home, in a nursing home) and on his/her awareness of dying (aware, not aware). The awareness is affected by the oncologist's communication about end of life.

The *family's preferred place of death* (home, hospital, nursing home) depends on the economic resources (type of health insurance, personal wealth and so on) available for caregiving, on the living area (rural, urban environment) and on the family suitability. A family is suitable if it is aware of the end-of-life (which again depends on the oncologist's communication), if at least a member of the family is available for home care and if he has high time availability.

The availability of a *palliative home-care* service depends on the general practitioner (available for home visits, no home visits) and on the type of cancer treatment.

Prior probabilities

Given a DAG, usually one learns a Bayesian network by estimating from data the conditional probability of each node given its parents, starting the learning process with a uniform prior over the parameter values.

To incorporate prior knowledge, we instead elicit informative priors from domain experts. Yet expert do not feel at their ease by reporting their knowledge as exact probability numbers. We thus allow them to express their judgments in natural language; later we translate them into intervals of probabilities as in [1], using the following dictionary: *maximal* (.8 - 1); *high* (.6- .8); *positive*(.4 -.6); *modest*(.2 -.4); *minimal* (0 - 0.2). We adopt the CREDO library [1] to implement the credal network. Some examples of such prior assessment for *place of death* are given in Tab.1.

**Parents configuration**				**Expert judgments**		
Pat. pref.	Fam. pref.	Hosp. days	Pall.	*Home*	*Nursing home*	*Hospital*
Hospital	Hospital	0-20	No	minimal	minimal	maximal
Home	Home	0-20	Yes	maximal	minimal	modest
Home	Home	0-20	No	high	minimal	modest

Table 1: Some prior intervals of *place of death*.

3 Experimental results

We validate the model by predicting the state of *place of death*, given the evidence about the remaining variables. In some hard-to-classify cases, the credal network returns multiple possible outcomes [1], thus highlighting its uncertainty.

Our anonymised data set regards 116 terminally ill cancer patients in southern Switzerland (age >20); it refers to the period 2014-2016. All patients deceased within a period of three months from the assessment. This was not a clinical trial, as we analyze retrospectively the data. For each patient we have the prediction about place of death issued by a team of experts We obtained the approval of the local Swiss ethical committee to proceed with data processing (BASEC Project ID 2016-01455).

Retrospectively, we make predictions about their place of death using leave-one-out cross-validation. We compare the credal network against the team of experts. As competing classifiers, we consider naive Bayes classifier (NBC) and averaged one-dependence estimator (AODE). These are standard classification algorithms; a description of both can be found for instance in [5].

The results are given in Tab. 2. The credal network identifies a single class as the most probable one in most cases (83%). On such instances, the credal network is more accurate (+4 accuracy points) than the team of experts but less

**Output of credal network**	**Proportion**	**Accuracies**			
		Credal	*Team*	*NBC*	*AODE*
One class	0.83	0.83	0.79	0.86	0.87
Two classes	0.16	1.00	0.53	0.53	0.53
Three classes	0.01	1.00	0.00	0.00	0.00

Table 2: Results obtained in predicting the place of death.

accurate (-4 accuracy points) than both NBC and AODE. This is nevertheless a positive result, considering the difficulties implied by learning such a large model (both NBC and AODE are more parameter-parsimonious).

Yet when the credal network returns two classes, both the team and the data mining classifiers undergo a sharp drop of accuracy (about -30 points). Thus the credal network has an important strength: it highlights the instances which are only partially predictable. In these cases it remains reliable by returning set-valued predictions.

The drop of accuracy of the competitors is even more extreme (accuracy 0) in the few cases in which the credal network returns all the three classes, acknowledging unpredictability. This is however a very small set of instances.

Such positive results are overall consistent with previous findings about the reliability of credal classifiers [3].

References

1. Antonucci, A., Huber, D., Zaffalon, M., Luginbuehl, P., Chapman, I., Ladouceur, R.: CREDO: a military decision-support system based on credal networks. In: Proceedings of the 16th Conference on Information Fusion (FUSION 2013). pp. 1–8 (2013)
2. Antonucci, A., Salvetti, A., Zaffalon, M.: Credal networks for hazard assessment of debris flows, pp. 125–132. Nova Science, Kropp, J., Scheffran, J., New York (2007)
3. Corani, G., Zaffalon, M.: Learning reliable classifiers from small or incomplete data sets: the naive credal classifier 2. Journal of Machine Learning Research 9, 581–621 (2008)
4. Cozman, F.G.: Credal networks. Artificial intelligence 120(2), 199–233 (2000)
5. Witten, I.H., Frank, E., Hall, M.A., Pal, C.J.: Data Mining: Practical machine learning tools and techniques. Morgan Kaufmann (2016)
