# Supplementary material for: Impact on place of death in cancer patients: a causal exploration in southern Switzerland
Source: BMC Palliat Care. 2020 Oct 15;19:160. doi: 10.1186/s12904-020-00664-4 (PMC7566155; doi:10.1186/s12904-020-00664-4)
Supplement: Supplementary file 4 — Additional file 4 : Table 3. Data collection procedure [file 12904_2020_664_MOESM4_ESM.pdf]

Additional file 4: Table 3. Data collection procedure

Table 3. Data collection (clinical chart review and review of doctors' and nurses' notes at a median of 38 days prior to death)		
Patient's awareness of dying*	0 closed<br>1 open<br>? not assessed	Closed: Patient avoided talking and/or asking questions about the prognosis or future perspectives<br>Open: Patient talked frankly about the poor prognosis or used metaphors indicating an adequate understanding of the situation
Patient's preference for the place of care at the end of life	0 Hospital<br>1 Home<br>2 Nursing home<br>? Unknown	Did the patient talk about preferences with the care team?
Treatment	0 Treatment discontinued<br>1 Ongoing active treatment	
Symptom burden	0 None or low<br>1 Medium<br>2 Severe	Visual analogue scale [VAS] 0-10 for symptoms such as pain, breathlessness, delirium, nausea, vomiting, constipation<br>None-low = VAS 0-2<br>Medium = VAS 2-6<br>High = VAS 6-10<br><br>High for unstable clinical conditions: life-threatening infections, bleeding, bowel obstruction, epileptic attacks, relevant neurological sequelae, organ dysfunction
Karnofsky Performance Status **	Number	* See table
Family's awareness of dying*	0 closed<br>1 open<br>? not assessed	Closed: family caregivers avoided talking and/or asking questions about the patient's prognosis or future perspectives<br>Open: talked frankly about the patient's poor prognosis or used metaphors indicating an adequate understanding of the situation
Family system's condition for home care	0 unsuitable condition<br>1 suitable condition	Unsuitable: No family caregiver available for home care or limited capacity for caregiving, not open to home visits and advice from home care services<br>Suitable: at least one caregiver available, good caregiving attitude, open to home visits and advice from professionals
Family's preference for the place of care at the end of life	0 Hospital<br>1 Home<br>2 Nursing home<br>? unknown	Did one of the primary family caregivers talk about preferences with anyone on the care team?
Care team's prediction of the place of death	0 Hospital<br>1 Home<br>2 Nursing home	Did the care team think the patient would die at home, in a hospital or in a nursing home?

* Adapted from the awareness of dying theory (Glaser and Strauss, 1965)

** Karnofsky Performance Status. European Society for Medical Oncology [ESMO]

100	Normal, no complaints
90	Able to carry on normal activity, minor signs or symptoms of disease
80	Normal activity with effort
70	Cares for self. Unable to carry on normal activity or to do active work
60	Requires occasional assistance, but able to care for most of his or her needs
50	Requires considerable assistance and frequent medical care
40	Disabled, requires special care and assistance
30	Severely disabled, hospitalisation is indicated although death non imminent
20	Very sick, hospitalisation necessary, active supportive treatment necessary
10	Moribund
0	Dead
