# Supplementary material for: Impact on place of death in cancer patients: a causal exploration in southern Switzerland
Source: BMC Palliat Care. 2020 Oct 15;19:160. doi: 10.1186/s12904-020-00664-4 (PMC7566155; doi:10.1186/s12904-020-00664-4)
Supplement: Supplementary file 5 — Additional file 5 : Table 4: Results from the causal probabilistic model and Table 5: Results from the classifier [file 12904_2020_664_MOESM5_ESM.pdf]

Additional file 5: Table 4. Results from the causal probabilistic model

Table 4: Results from the causal probabilistic model

Queries (Q)	Target variables and outcome probabilities %		
	Outcome classes of the target variable Cancer treatment		
Q 1. Impact of cancer treatment resources on Cancer treatment	***Ongoing active treatment***		***Treatment discontinued***
Treatment resources available	0.640-0.800		0.200-0.360
No treatment resources available	0.040-0.272		0.728-0.960
	Outcome classes of the target variable Communication of poor prognosis		
Q 2. Impact of cancer treatment on Communication	***Little information about the end of life given***	***Partial information given***	***Full information given***
Cancer treatment resources available, ongoing active treatment	0.000-0.200	0.600-0.800	0.200-0.400
No treatment resources available, treatment discontinued	0.000-0.000	0.400-0.400	0.600-0.600
	Outcome classes of the target variable Family's awareness of dying		
Q 3. Impact of palliative chemotherapy and communication on Family's awareness of dying	***Closed awareness***		***Open awareness***
Cancer treatment resources available, ongoing active treatment, little information given about poor prognosis	0.600-0.800		0.200-0.400
No treatment resources available, treatment discontinued, full information about the end of life given	0.200-0.400		0.600-0.800
	Outcome classes of the target variable Patient's awareness of dying		
Q 4. Impact of palliative chemotherapy and communication on Patient's awareness of dying	***Closed awareness***		***Open awareness***
Cancer treatment resources available, ongoing active treatment, little information given about poor prognosis	0.800-0.800		0.200-0.200
No treatment resources available, treatment discontinued, full information about the end of life given	0.200-0.400		0.600-0.800
	Outcome classes of the target variable Hospital days		
Q 5. Impact of chemotherapy on Hospital days	***0-20***	***21-40***	***41-60***
Cancer treatment resources available, ongoing active treatment	0.640-0.794	0.206-0.360	0.000-0.092
No treatment resources available, treatment discontinued	0.800-0.924	0.076-0.200	0.000-0.124
	Outcome classes of the target variable Hospital days		
Q 6. Impact of symptom burden on Hospital days	***0-20***	***21-40***	***41-60***
No-low symptom burden	0.800-0.987	0.013-0.200	0.000-0.187
Medium-high symptom burden	0.660-0.791	0.209-0.340	0.000-0.060
	Outcome classes of the target variable POD		
Q 7. Impact of disease-related variables on POD	***Home***	***Nursing Home***	***Hospital***
Cancer treatment resources available, ongoing active treatment, medium-high symptom burden, 41-60 hospital days, little information given, closed awareness of dying among patient and family	0.087-0.351	0.025-0.278	0.569-0.888
No treatment resources available, treatment discontinued, no-low symptom burden, 0-20 hospital days, full information about the end of life given, open awareness among patient and family	0.213-0.575	0.025-0.282	0.336-0.664
	Outcome classes of the target variable Patient's preference for place of care		
Q 8. Patient's preference for place of care	***Home***	***Nursing home***	***Hospital***
Patient's residence at home	0.374-0.633	0.063-0.143	0.278-0.530
Patient's residence at nursing home	0.230-0.307	0.571-0.676	0.079-0.143
	Outcome classes of the target variable Family's preference for place of care		
Q 9. Family's preference for place of care	***Home***	***Nursing home***	***Hospital***
Urban environment	0.158-0.492	0.017-0.218	0.481-0.816
Rural environment	0.349-0.600	0.026-0.143	0.374-0.625

Table 4: Results from the causal probabilistic model (continued)

Queries (Q)	Target variables and outcome probabilities %		
	Outcome classes of the target variable POD		
Q 10. Impact of family's preference for place of care on POD	***Home***	***Nursing home***	***Hospital***
Family's preference for home	0.538-0.698	0.013-0.119	0.282-0.437
Family's preference for hospital	0.057-0.283	0.014-0.203	0.587-0.873
Family's preference for nursing home	0.000-0.199	0.503-0.795	0.205-0.497
	Outcome classes of the target variable POD		
Q 11. Impact of patient's and family's preferences for place of care on POD	***Home***	***Nursing home***	***Hospital***
Family's preference for home and patient's preference for home	0.636-0.759	0.000-0.050	0.241-0.364
Family's preference for hospital and patient's preference for home	0.156-0.360	0.000-0.180	0.497-0.753
Family's preference for nursing home and patient's preference for home	0.000-0.200	0.600-0.800	0.200-0.400
Family's preference for home and patient's preference for hospital	0.481-0.600	0.000-0.180	0.400-0.508
	Outcome classes of the target variable Family system		
Q 12. Impact of correlations of family-related variables on Family system	***Suitable conditions for home care***	***Unsuitable conditions for home care***	
At least one person available for home care, open awareness of dying among family, somewhat solid family emotional relationship, patient almost independent, full information about the end of life given	0.800-1.000	0.000-0.200	
Nobody available for home care, closed awareness of dying, somewhat conflictual family emotional relationship, patient needs continuous help, partial information about end of life given	0.000-0.200	0.800-1.000	
At least one person available for home care, somewhat solid family emotional relationship, patient almost independent, full information about the end of life given, closed awareness of dying	0.400-0.400	0.600-0.600	
Nobody available for home care, somewhat solid family emotional relationship, patient almost independent, full information about the end of life given, open awareness of dying	0.200-0.400	0.600-0.800	
	Outcome classes of the target variable Family's availability for home care		
Q13. Impact of dependence degree and symptom burden on Family's availability for home care	***At least one person available***	***Nobody available***	
Patient almost independent, low symptom burden, open awareness of dying among family, somewhat solid family emotional relationship, full information about the end of life given	0.800-1.00	0.000-0.200	
Patient needs continuous help, medium-high symptom burden, open awareness of dying among family, somewhat solid emotional relationship, full information about the end of life given	0.400-0.400	0.600-0.600	
	Outcome classes of the target variable POD		
Q 14. Impact of all family-related variables on POD	***Home***	***Nursing home***	***Hospital***
Patient needs continuous help, high symptom burden, somewhat conflictual family emotional relationship, closed awareness of dying among family, unsuitable conditions for home care, poor time resources, nobody available, distance range >50 km,	0.105-0.433	0.026-0.297	0.461-0.807
Patient almost independent, low symptom burden, somewhat solid family emotional relationship, open awareness of dying among family, suitable conditions for home care, unlimited time resources, distance range 0-5 km	0.239-0.535	0.025-0.267	0.388-0.685

**Table 4**: Results from the causal probabilistic model (continued)			
Queries (Q)	Target variables and outcome probabilities %		
	Outcome classes of the target variable Family's preference for place of care		
Q 15. Impact of economic resources and family system's conditions on Family's preference	***Home***	***Nursing home***	***Hospital***
Patient's residence at home, patient's preference for home, patient needs continuous help, family system's suitable conditions for home care, poor economic resources	0.433-0.600	0.000-0.167	0.400-0.567
Patient's residence at home, patient's preference for home, patient needs continuous help, family system's suitable conditions for home care, high economic resources	0.433-0.667	0.000-0.200	0.333-0.567
Patient's residence at home, patient's preference for home, patient needs continuous help, family system's unsuitable conditions for home care, poor economic resources	0.033-0.267	0.000-0.200	0.733-0.967
Patient's residence at home, patient's preference for home, patient needs continuous help, family system's unsuitable conditions for home care, high economic resources	0.233-0.400	0.000-0.167	0.600-0.767
	Outcome classes of the target variable POD		
Q 16. Interventional test of the impact of health care-related variables on POD	***Home***	***Nursing home***	***Hospital***
Home care costs fully covered by insurance and health care policy, GP home visits, specialist palliative home care service, continuous professional home care, volunteer hospice service 24/7	0.176-0.543	0.026-0.294	0.359-0.704
Home care costs partially covered by insurance and health care policy, no GP home visits, no specialist palliative home care service, no professional home care, volunteer hospice service available a few hours	0.113-0.404	0.025-0.269	0.528-0.833
Patient's residence at home, patient's and family's preferences for home, home care costs fully covered by insurance, GP home visits, specialist palliative home care service, routine home care, volunteer hospice service 24/7	0.759-0.827	0.000-0.062	0.173-0.241
	Outcome classes of the target variable POD		
Q 17. Interventional test of the impact of health care-related variables and preferences on POD	***Home***	***Nursing home***	***Hospital***
Patient's residence at home, patient's and family's preferences for home, home care costs fully covered by insurance and health care policy, GP home visits, routine home care, volunteer hospice service, no specialist palliative home care service	0.503-0.592	0.000-0.035	0.408-0.497
Patient's residence at home, patient's and family's preferences for home, home care costs fully covered by insurance and health care policy, GP home visits, continuous home care, volunteer hospice service, no specialist palliative home care service	0.503-0.592	0.000-0.035	0.408-0.497
Patient's residence at home, patient's preference for home, family's preference for hospital, home care costs covered by insurance and health care policy, GP home visits, specialist palliative home care service, continuous home care, volunteer hospice service 24/7	0.189-0.400	0.000-0.200	0.400-0.621
	Outcome classes of the target variable Family's preference for place of care		
Q 18. Interventional test of the impact of modifiable variables on Family's preference	***Home***	***Nursing home***	***Hospital***
Patient's residence at home, GP home visits, specialist palliative home care service, continuous assistance by a home care service, home care costs fully covered by insurance and health care policy, availability of a volunteer hospice service 24/7	0.213-0.549	0.000-0.186	0.451-0.787
Patient's residence at home, little information given, no GP home visits, no specialist palliative home care service, no home care frequency, home care costs partially covered by insurance and health care policy, availability of a volunteer hospice service a few hours	0.197-0.540	0.000-0.185	0.460-0.803

**Table 4**: Results from the causal probabilistic model (continued)		
Queries (Q)	Target variables and outcome probabilities %	
	Outcome classes of the target variable Family system	
Q 19. Interventional test of the impact of communication, awareness of dying, symptom burden on the Family system	***Suitable conditions for home care***	***Unsuitable conditions for home care***
Patient's residence at home, little information given (action), closed awareness of dying among patient and family, high symptom burden	0.104-0.293	0.707-0.896
Patient's residence at home, full information about the end of life given (action), open awareness of dying among patient and family, low symptom burden	0.459-0.864	0.136-0.541

Table 5: Results from the classifier

**Table 5**: Results from the classifier			
States of variables	Prediction for Place of death (%)		
	Home	***Hospital***	***Nursing home***
a) Patient's residence at home, 0-20 hospital days, rural environment, low symptom burden, KPS 45-65, routine home care frequency, GP home visits, specialist palliative home care service provided, open awareness among patient and family, both family and patient preferences for home as place of care, family system's suitable conditions	0.9681	0.0318	0.0001
b) Same states as in test a) but in *urban environments*	0.824	0.1753	0.0007
c) Same states as in test b) but *palliative home care service not provided*	0.4594	0.539	0.0016
d) Patient's residence at home, urban environment, KPS 45-65, routine home care frequency, GP home visits, specialist palliative home care service provided, open awareness of dying among patient and family, both family and patient preferences for home as place of care, family system's suitable conditions, 0-20 hospital days, *medium-high symptom burden*	0.5874	0.4124	0.0002
e) Patient's residence at home, urban environment, KPS 45-60, routine home care frequency, GP home visits, specialist palliative home care service provided, open awareness of dying among patient and family, both family and patient preferences for home as place of care, family system's suitable conditions, *41-60 hospital days, medium-high symptom burden*	0.2336	0.7651	0.0014
f) Patient's residence at home, 0-20 hospital days, urban environment, low symptom burden, KPS 45-65, routine home care frequency, GP home visits, specialist palliative home care service provided, open awareness of dying among patient and family, both family and patient preferences for home as place of care, *family system's unsuitable conditions*	0.4655	0.5219	0.0126
g) Same states as in test f) but *closed awareness of dying among family*	0.1267	0.8449	0.0284
h) Patient's residence at home, 0-20 hospital days, urban environment, KPS 45-65, routine home care frequency, GP home visits, specialist palliative home care service provided, low symptom burden, patient's open awareness of dying, *unknown preferences of the patient and the family, unknown family system conditions, unknown state of awareness of the family*	0.359	0.5842	0.0568
i) Same states as in test h) but *medium-high symptom burden*	0.0915	0.9038	0.0047
